# The CD73-adenosine axis in melanoma: from multidimensional regulation to precision immunotherapy

**DOI:** 10.3389/fimmu.2026.1839832

**Published:** 2026-07-30

**Authors:** Xiaoting Liu, Pengcheng Sun, Can Chen, Xi Chen

**Affiliations:** 1 Huzhou Central Hospital, Fifth School of Clinical Medicine of Zhejiang Chinese Medical University, Huzhou, China; 2 Huzhou Central Hospital, Affiliated Central Hospital of Huzhou University, Huzhou, China; 3 Soochow University, Suzhou, China; 4 Department of Dermatology, First Affiliated Hospital of Huzhou University, Huzhou, China

**Keywords:** adenosine, CD73, immunotherapy, melanoma, tumor microenvironment

## Abstract

The therapeutic landscape of advanced melanoma has been transformed by immune checkpoint inhibitors (ICIs); however, primary and acquired resistance remain formidable challenges for a substantial subset of patients. Emerging evidence identifies the CD73-adenosine axis as a critical “metabolic immune checkpoint” that orchestrates an immunosuppressive tumor microenvironment (TME). This review systematically explores the biochemical cascade in which extracellular ATP is converted into immunosuppressive adenosine by the sequential action of ectonucleotidases CD39 and CD73. We detail the multidimensional regulation of CD73 expression, driven by a convergence of environmental stressors such as hypoxia-stabilized HIF-1α, oncogenic MAPK signaling, and pro-inflammatory cytokines including TGF-β and TNF-α. Furthermore, we evaluate the clinical utility of CD73 as a prognostic and predictive biomarker, highlighting the dichotomous significance of its expression on tumor versus immune cells and the potential of liquid biopsy-based monitoring via soluble CD73 activity and melanoma-derived exosomes (MTEX). Finally, we discuss therapeutic strategies to dismantle this metabolic barrier, ranging from monoclonal antibodies and small-molecule inhibitors to synergistic combinations with ICIs, targeted therapies, and next-generation cellular engineering utilizing CRISPR/Cas9-mediated gene editing. Integrating adenosinergic blockade into the framework of precision immuno-oncology represents a pivotal dimension in overcoming treatment resistance and improving clinical outcomes for patients with melanoma.

## 1 Introduction

Cutaneous melanoma represents a significant public health burden due to its rising incidence and potential for metastatic spread and mortality (1–3). Historically associated with a dismal prognosis, the treatment landscape for advanced melanoma has undergone a revolutionary transformation over the past decade (4). This paradigm shift is largely attributed to the clinical success of immune checkpoint inhibitors (ICIs), which have redefined therapeutic standards and patient outcomes (5, 6). Therapeutic agents targeting the cytotoxic T-lymphocyte-associated antigen 4 (CTLA-4) and programmed cell death 1 (PD-1) axes have demonstrated remarkable efficacy, producing durable responses in a substantial subset of patients (4). The approval of these agents was predicated on the foundational understanding that melanoma is among the most immunogenic solid tumors, possessing a high tumor mutational burden and an inflamed tumor microenvironment (TME) that should, in principle, be receptive to immune reactivation (7).

The clinical validation of immunotherapy has firmly established the blockade of co-inhibitory immune checkpoints as a cornerstone of modern oncology (5, 6). By disrupting the PD-1/PD-L1 and CTLA-4 pathways, these therapies release pre-existing anti-tumor T cells from inhibitory signals, leading to tumor regression. This approach has moved from a salvage therapy for metastatic disease to an adjuvant and neoadjuvant strategy, significantly improving long-term survival rates (4, 8). However, the celebration of this success is tempered by a sobering reality. Despite these advances, a significant proportion of patients with advanced melanoma derive no clinical benefit from ICI monotherapy or combination regimens (8, 9). A considerable number of patients exhibit primary (innate) resistance, failing to respond from the outset. Conversely, others who initially respond eventually relapse due to acquired (adaptive) resistance, underscoring the multifaceted nature of tumor immune evasion (8, 10, 11). This highlights a critical unmet need: overcoming the myriad immunosuppressive mechanisms that coexist within the TME to restore effective anti-tumor immunity in non-responders and prevent relapse in responders (8, 11).

Research efforts have therefore intensified to decipher the molecular and cellular underpinnings of ICI resistance (8). It is now recognized that tumor immune escape is orchestrated by a network of interconnected, non-redundant pathways that extend beyond the canonical PD-1 and CTLA-4 checkpoints (10, 12). Among the extrinsic factors contributing to the immunosuppressive TME, metabolic antagonism has emerged as a pivotal barrier to effective immunotherapy (8, 13). The hostile metabolic milieu of solid tumors, characterized by nutrient deprivation, hypoxia, and accumulation of waste products, directly impairs the function of infiltrating immune effector cells while favoring pro-tumorigenic and regulatory populations (14–16). A key immunosuppressive metabolite in this context is extracellular adenosine. Within the hypoxic core of tumors, the breakdown of extracellular ATP, released from stressed and dying cells, leads to the accumulation of adenosine via the sequential action of ectonucleotidases CD39 and CD73 (13, 17, 18).

This hypoxia-adenosinergic pathway functions as a potent, non-redundant immunosuppressive circuit (15, 17). Adenosine engages high-affinity A2A receptors (A2ARs) and A2B receptors (A2BRs) on immune cells, triggering intracellular cyclic AMP-mediated signaling that broadly suppresses effector functions of T cells and natural killer (NK) cells, while simultaneously promoting the expansion and activity of regulatory T cells (Tregs) and myeloid-derived suppressor cells (MDSCs) (10, 15, 17). Beyond this canonical surface receptor engagement, recent breakthroughs have unveiled a critical intracellular dimension to adenosine-mediated immunosuppression. Emerging studies demonstrate that extracellular adenosine can also be transported across the cell membrane through nucleoside transporters. Specifically, T cells upregulate the equilibrative nucleoside transporter-1 (ENT1) upon activation, which serves as the major regulator facilitating active adenosine uptake. Once internalized, this intracellular adenosine profoundly impairs T cell expansion and effector functions by inhibiting *de novo* pyrimidine nucleotide synthesis, ultimately causing pyrimidine starvation. Conversely, blocking or deleting ENT1 protects T cells from this metabolic suppression, significantly enhancing CD8+ T-cell-dependent antitumor responses and synergizing with PD-1 blockade. This intracellular metabolic interference adds a highly complex and non-redundant layer to the ADO-induced immunosuppressive network (19, 20). In melanoma, accumulating evidence suggests that dysregulated adenosine signaling is not merely a bystander but an active driver of pathogenesis, influencing tumor cell proliferation, invasion, and angiogenesis while crippling the host immune response (13, 21). The CD73-adenosine axis has thus been conceptualized as a critical “metabolic immune checkpoint,” acting in concert with, but independently of, PD-1/PD-L1 and CTLA-4 to enforce an immunosuppressive state (12, 15).

Consequently, therapeutic targeting of the adenosinergic pathway represents a compelling and rational strategy to expand the benefits of immunotherapy to a broader patient population (10, 12). Preclinical studies in melanoma and other cancers have demonstrated that inhibiting adenosine generation via anti-CD73 or anti-CD39 antibodies, or blocking adenosine receptor signaling with selective antagonists, can reinvigorate anti-tumor immunity and synergize with existing ICIs (13, 17, 21). This has spurred the rapid clinical development of a new class of immunotherapeutic agents aimed at dismantling this metabolic barrier (10, 18). The emergence of the CD73-adenosine axis as a novel target marks a significant evolution in the field of melanoma immunotherapy, shifting the focus from overcoming singular immune-inhibitory receptors to reprogramming the broader immunosuppressive metabolic landscape of the TME.

To ensure a comprehensive and rigorous synthesis of the current evidence, a systematic literature search was conducted. We searched electronic databases including PubMed, Web of Science, and Scopus for peer-reviewed articles published from inception to May 2026. The search strategy employed Boolean operators with the following primary keywords: (“Melanoma” OR “Cutaneous Melanoma”) AND (“CD73” OR “NT5E” OR “Ecto-5’-nucleotidase”) AND (“Adenosine” OR “Purinergic Signaling”) AND (“Tumor Microenvironment” OR “Immunotherapy” OR “Immune Checkpoint”). Articles were independently screened based on their relevance to the molecular regulation, prognostic value, and therapeutic targeting of the CD73-adenosine axis in melanoma. Following the exclusion of duplicates and non-relevant publications, a total of 154 articles were selected and included in this review to construct the conceptual framework.

Unlike previous literature that broadly outlines purinergic signaling, the distinct novelty of this review resides in its compartment-specific analysis of CD73’s prognostic value and its deep integration with emerging clinical applications. We systematically bridge the fundamental biochemistry of the CD73-adenosine axis with cutting-edge non-invasive liquid biopsy modalities—specifically soluble CD73 catalytic activity and melanoma-derived exosomes (MTEX)—providing a mechanism-driven roadmap for identifying early acquired resistance to anti-PD-1 therapies. Ultimately, this work offers a highly translational perspective on dismantling the immunosuppressive metabolic barrier to advance precision immuno-oncology for patients with melanoma.

## 2 The CD73-adenosine pathway: molecular players, biochemical regulation, and physiological context

**Figure 1:**
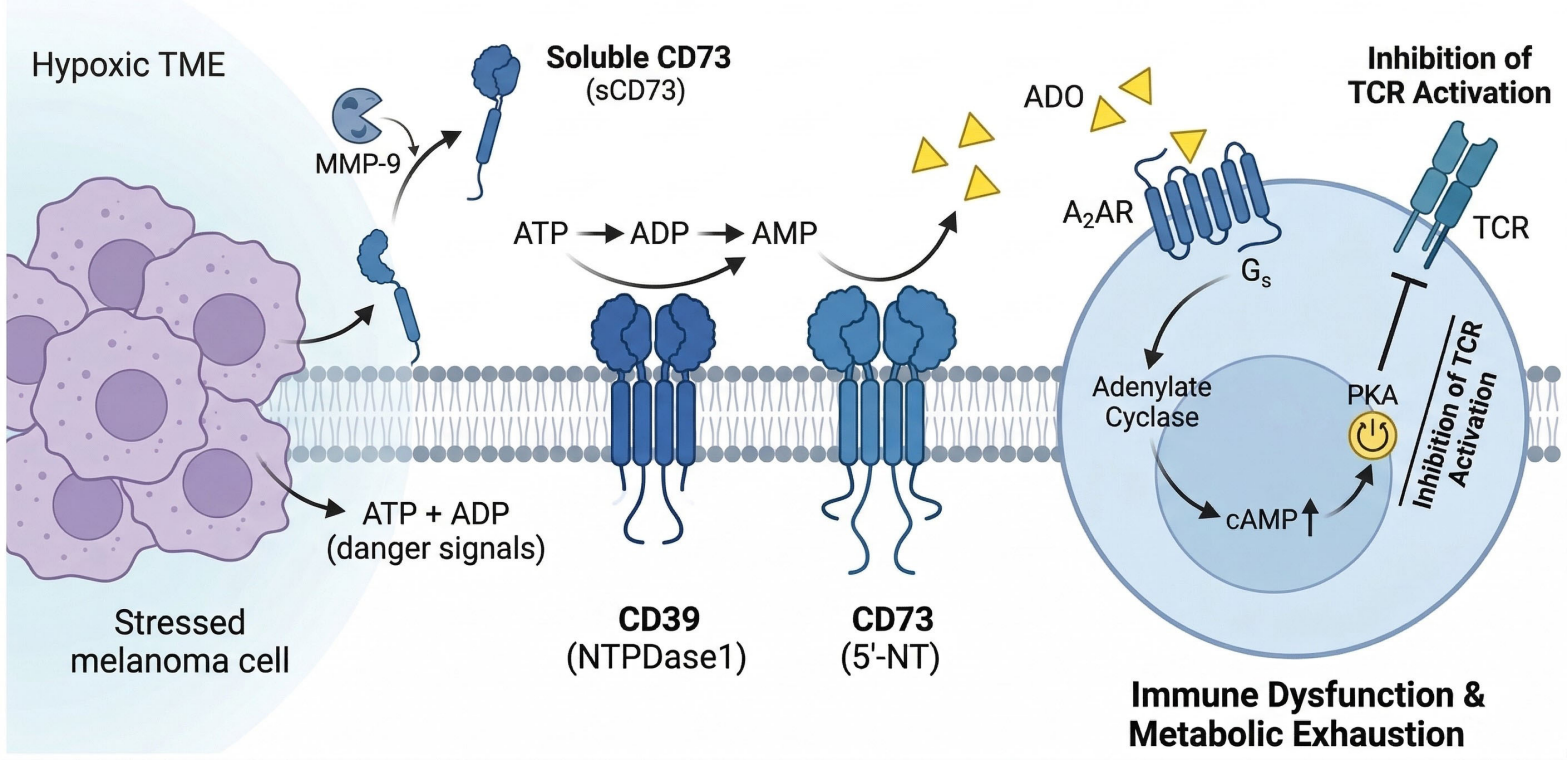
The CD39-CD73-adenosine purinergic signaling axis in the hypoxic melanoma microenvironment. Under hypoxic stress, melanoma cells release ATP and ADP into the extracellular space as danger-associated molecular patterns. CD39 (NTPDase1) initiates the cascade by hydrolyzing these nucleotides into AMP, which is subsequently converted into ADO by CD73 (5’-NT). MMP-9 mediates the proteolytic shedding of membrane-bound CD73 to generate catalytically active soluble CD73 (sCD73). Extracellular adenosine binds to the high-affinity A2AR on effector immune cells, triggering a G-protein/Adenylate Cyclase/cAMP/PKA canonical signaling pathway. This cascade potently suppresses T-cell receptor (TCR) activation, ultimately culminating in profound immune dysfunction and metabolic exhaustion of tumor-infiltrating lymphocytes.

The rapid clinical development of agents targeting the adenosinergic pathway is built upon a sophisticated understanding of its molecular architecture and the intricate biochemical networks it regulates (22, 23). At its core, this pathway functions as a powerful rheostat for extracellular purinergic signaling, converting immunostimulatory nucleotides into potent immunosuppressive nucleosides, a process profoundly dysregulated within the TME (24, 25). The canonical pathway initiates with the release of ATP, often from stressed or dying cells, into the extracellular space where it serves as a potent danger signal, activating P2 purinergic receptors on immune cells to promote inflammation and immune cell activation (26, 27). This pro-inflammatory state is tightly controlled by the sequential action of two key ectoenzymes. CD39 (ectonucleoside triphosphate diphosphohydrolase-1, NTPDase1) acts as the gatekeeper, catalyzing the hydrolysis of ATP and ADP to AMP (28, 29). The final and committed step is executed by CD73 (ecto-5’-nucleotidase), a glycosylphosphatidylinositol (GPI)-anchored protein that dephosphorylates AMP to generate adenosine (ADO) (30, 31) (**Figure 1**). The biochemical properties and regulation of these enzymes, particularly CD73, are therefore central to controlling the balance between pro-inflammatory ATP and immunosuppressive adenosine (32). In summary, the CD39-CD73 biochemical cascade functions as a sophisticated metabolic rheostat, decisively shifting the TME from an immunostimulatory to an immunosuppressive state. However, a major shortcoming in current research is the incomplete understanding of purinergic redundancy. While the canonical CD39/CD73 axis is well-characterized, alternative adenosine-generating pathways (such as the CD38/PC-1 axis) remain insufficiently mapped in the clinical setting. This redundancy poses a significant challenge, as inhibiting a single ectoenzyme may inevitably lead to compensatory activation of alternative pathways, highlighting a critical gap in our current strategy for complete adenosinergic blockade.

CD39 is a type-II transmembrane protein whose ectodomain contains apyrase conserved regions critical for its nucleotidase activity, requiring divalent cations like Ca²^+^ or Mg²^+^ for function (33, 34). While CD39 is frequently characterized as an initial rate-limiting enzyme because its expression and specific activity dictate the initial flux of substrates into the purinergic pathway, this regulatory dynamic is highly nuanced. Specifically, the extracellular hydrolysis of ATP to AMP by CD39 is somewhat reversible due to the presence of extracellular kinases. In contrast, the subsequent dephosphorylation of AMP to adenosine by CD73 is an essentially irreversible, committed step. Consequently, both ectonucleotidases function as distinct rate-limiting bottlenecks in the cascade: CD39 governs the initial substrate influx, whereas CD73 dictates the terminal, irreversible generation of immunosuppressive adenosine (26, 35). Its localization on endothelial cells, Tregs, and certain myeloid subsets positions it strategically within tissues (28, 36). In contrast, CD73 is a GPI-anchored homodimer that facilitates the terminal conversion (31, 37). Beyond its enzymatic role, CD73 can also function as an adhesion molecule, interacting with components of the extracellular matrix and other cell-surface proteins, which may influence lymphocyte trafficking and cell-cell interactions within tissue niches (37, 38). The catalytic mechanism of CD73 has been studied using various substrates and inhibitors, revealing important insights for drug development (30, 39). The enzymatic product of this coordinated effort, adenosine, exerts its biological effects by engaging four G-protein coupled receptors (GPCRs): A_1_, A_2_A, A_2_B, and A_3_ (23, 40). Each receptor subtype has distinct expression patterns, ligand affinities, and downstream signaling cascades. The A2AR, a high-affinity receptor for adenosine, is coupled to Gαs proteins, leading to stimulation of adenylate cyclase, elevated intracellular cyclic AMP (cAMP), and activation of protein kinase A (PKA) (41, 42). This cAMP-PKA axis is a master regulator of immune suppression, inhibiting T cell receptor (TCR) signaling, cytokine production (e.g., IFN-γ, granzyme B), and proliferation in CD8+ T cells and NK cells (41, 43). The A2AR can also cross-talk with other critical pathways, such as inhibiting TCR-induced Notch1 activation, a transcription factor required for optimal CD8+ T cell function (41). The lower-affinity A2BR is coupled to both Gαs and Gαi proteins and becomes significant under pathological conditions where adenosine concentrations are high, such as in hypoxic tumors (40, 44). A2BR signaling on dendritic cells can promote an immunosuppressive phenotype, including the secretion of IL-6 and IL-10, which can skew T cell differentiation towards pro-tumorigenic T helper 17 (Th17) cells or contribute to a tolerogenic environment (45, 46). The roles of A_1_ and A_3_ receptors in cancer are more context-dependent but can influence cell proliferation and survival in certain settings (47, 48).

The expression and activity of the adenosinergic pathway are dynamically regulated by central microenvironmental cues. Hypoxia, a hallmark of solid tumors, is a potent inducer via the transcription factor hypoxia-inducible factor-1α (HIF-1α) (49, 50). HIF-1α can directly transactivate genes like CD73 and ADORA2B (encoding A2BR), creating a feed-forward loop where hypoxia increases both the production and sensing of adenosine (44). The immunosuppressive cytokine transforming growth factor-beta (TGF-β) is another critical regulator, shown to induce CD73 expression on CD4+ T cells, CD8+ T cells, dendritic cells, and macrophages, thereby amplifying adenosine generation capacity in a contextual manner (32, 51). This induction operates at the transcriptional level and is independent of Foxp3, linking TGF-β signaling directly to purinergic immunosuppression. Furthermore, adenosine itself can modulate the expression of its own machinery; chronic adenosine exposure can influence the surface expression of molecules on antigen-presenting cells and enhance the ectoenzymatic network (46, 52). This complex regulation ensures that the adenosinergic pathway is exquisitely responsive to tissue states, acting as a homeostatic mechanism to prevent excessive inflammation and collateral damage under physiological conditions (53, 54). In normal physiology, this pathway is crucial for maintaining immune tolerance, dampening immune responses after the clearance of pathogens, and protecting tissues during events like ischemia-reperfusion injury (23, 55, 56).

However, in the pathological context of cancer, this protective mechanism is co-opted by tumors to establish an immunosuppressive niche. The TME is characterized by hypoxia, cell death, and high levels of TGF-β, all of which conspire to upregulate the CD39/CD73 axis and adenosine receptors on both tumor cells and infiltrating host cells (22, 25). Beyond the canonical CD39-CD73 axis, alternative pathways for adenosine generation exist and may be relevant in specific contexts. For instance, the ectoenzymes CD38 (an NAD+ glycohydrolase) and PC-1 (CD203a, a nucleotide pyrophosphatase/phosphodiesterase) can initiate a non-canonical pathway from NAD+ to ADO via ADPR and AMP intermediates (57, 58). This pathway has been demonstrated to be functional in primary human melanoma cells, contributing to the suppression of T cell proliferation (57). The coordinated regulation of these enzymes, often observed upon T cell activation, suggests a broader purinergic network for nucleotide recycling and immune modulation (52). The resultant adenosine accumulation engages primarily A2AR and A2BR on effector T cells and NK cells, directly suppressing their cytotoxic functions, cytokine production, and proliferation (43, 59). Concurrently, adenosine signaling on other immune cells, such as dendritic cells and MDSCs, promotes the secretion of anti-inflammatory cytokines like IL-10 and VEGF, further shaping an immunosuppressive TME (45, 46, 50). This multifaceted suppression positions the CD73-adenosine axis as a dominant metabolic checkpoint in cancer, functionally parallel to and often co-expressed with other checkpoint pathways like PD-1/PD-L1 (53, 60, 61). The biochemical consequence of this axis is not merely the accumulation of adenosine but also the depletion of extracellular ATP, which itself has direct anti-proliferative and pro-inflammatory effects mediated through P2 receptors, particularly P2X7 (27, 36). Thus, CD39 activity promotes tumor growth not only by generating immunosuppressive adenosine but also by scavenging immunostimulatory ATP, a dual mechanism that underscores its pivotal role (24, 36). The intricate interplay between these molecular players, their regulation by the TME, and their profound impact on immune cell function form the foundational rationale for targeting this axis to overcome a major barrier to effective anti-tumor immunity.

## 3 Regulation and functional significance of CD73 expression in the melanoma ecosystem

The immunosuppressive adenosine-rich niche, a consequence of upregulated CD39/CD73 activity, does not arise stochastically but is actively sculpted by the melanoma cells themselves and the hostile conditions of the TME. CD73 expression on both malignant and stromal cells is dynamically regulated by a network of cell-intrinsic oncogenic pathways and extrinsic microenvironmental cues, which in turn dictates its multifaceted functional significance in promoting tumor progression, immune evasion, and therapeutic resistance. Transcriptional regulation is a key layer of control. The homeodomain transcription factor HOXD13 drives a melanoblast-like developmental program that is upregulated in melanoma and strongly correlated with angiogenesis and immune exclusion (62, 63). HOXD13 orchestrates long-range chromatin contacts to simultaneously activate the expression of VEGFA, SEMA3A, and CD73, thereby coupling vascular remodeling with adenosinergic immunosuppression (62, 63). This coordinated regulation positions CD73 as an integral component of a broader transcriptional network promoting an immune-desert TME.

Epigenetic mechanisms, particularly DNA methylation and microRNA (miRNA) activity, provide another crucial regulatory layer for NT5E (the gene encoding CD73). In melanoma, the NT5E CpG island can be subject to methylation-dependent transcriptional silencing, and loss of this methylation—leading to CD73 upregulation—is associated with more aggressive disease and metastasis to visceral sites and the brain (64). Conversely, specific miRNAs potently modulate CD73 expression in melanoma cell lines. MiR-1285-5p, miR-155-5p, and miR-3134 were found to be potent inhibitors of NT5E, while miR-134-3p, miR-6859-3p, miR-6514-3p, and miR-224-3p strongly enhanced its expression (65). This miRNA-mediated regulation suggests a complex post-transcriptional control system where dysregulation can directly impact the adenosinergic landscape of the tumor.

Oncogenic signaling pathways central to melanoma pathogenesis directly govern CD73 expression. The MAPK pathway, frequently hyperactivated through BRAF mutations, is a major driver (66, 67). Activating MAPK mutations, along with growth factors, can induce CD73 expression, which is often linked to a mesenchymal-like phenotype switch in melanoma cells (66). Importantly, this pathway also offers a therapeutic vulnerability; treatment with BRAF inhibitors (dabrafenib, vemurafenib) or MEK inhibitors (trametinib) profoundly downregulates CD73 expression in BRAF-mutant melanoma cells (68, 69). This downregulation is part of the therapeutic effect, as combining BRAF/MEK inhibition with adenosine receptor blockade provided superior anti-tumor and anti-metastatic activity in preclinical models (68). Furthermore, the inflammatory cytokine TNF-α can cooperate with MAPK signaling via the c-Jun/AP-1 transcription factor complex to activate CD73 transcription through an intronic enhancer, linking inflammatory signals within the TME to enhanced immunosuppressive metabolite production (66).

**Figure 2:**
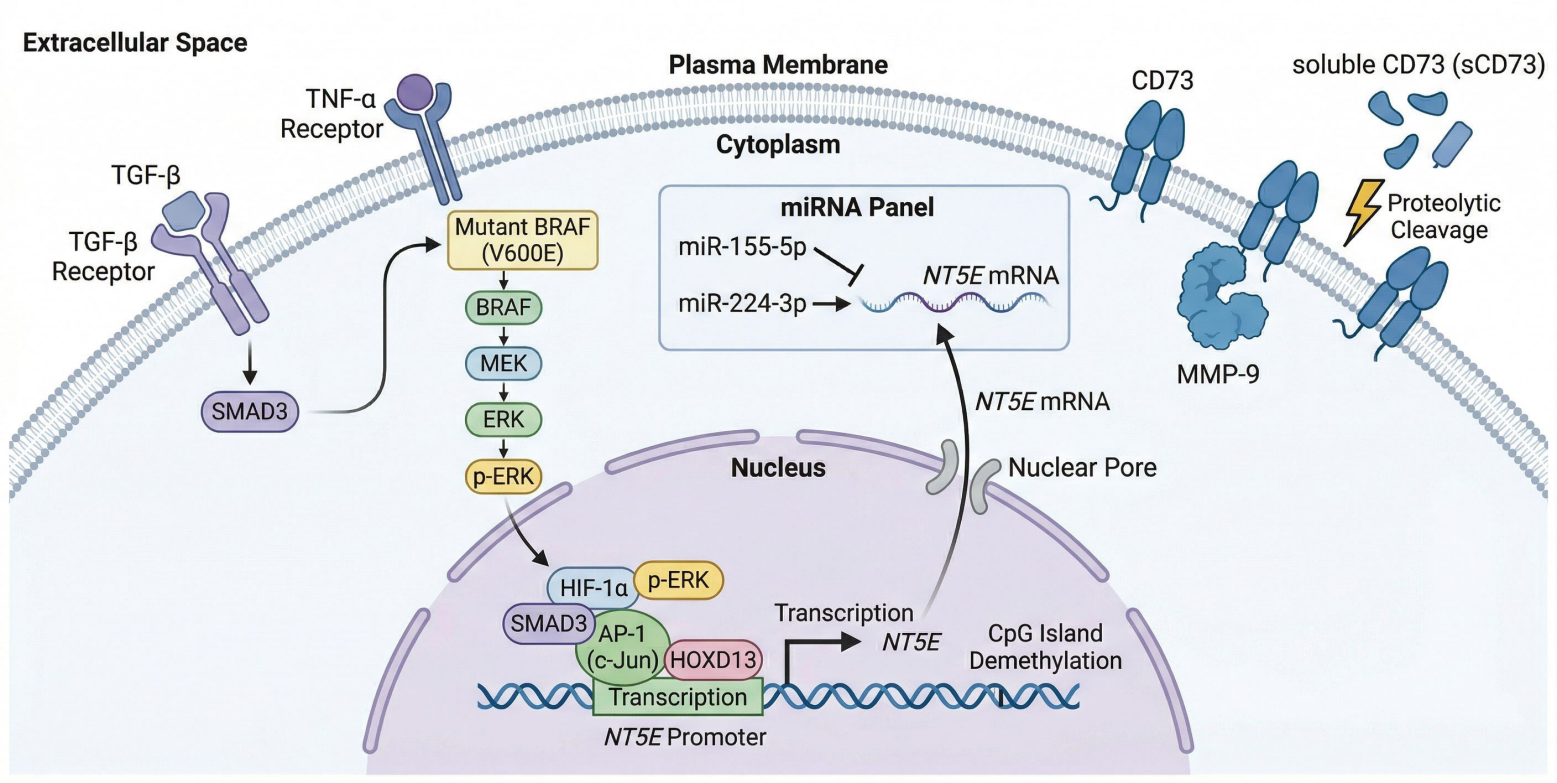
Multidimensional regulation of CD73 (NT5E) in the melanoma ecosystem. Hypoxia-stabilized HIF-1α, TGF-β, and TNF-α cooperate with the mutant BRAF-activated MAPK/ERK pathway to recruit a transcriptional complex comprising p-ERK, SMAD3, AP-1 (c-Jun), and HOXD13 to the NT5E promoter, a process further facilitated by CpG island demethylation. Post-transcriptionally, NT5E mRNA is regulated by miR-155-5p and miR-224-3p within the cytoplasm. Finally, membrane-bound CD73 homodimers undergo MMP-9-mediated proteolytic cleavage, releasing catalytically active sCD73 into the extracellular tumor.

The harsh TME itself is a potent inducer of CD73. Hypoxia, a near-universal feature, upregulates CD73 expression in a HIF-1α-dependent manner (70). Systemic oxygenation in mouse models decreased TME hypoxia, HIF-1α levels, and the expression of CD73 and CD39, thereby lowering extracellular adenosine and enhancing tumor immune recognition (70). Nutrient deprivation, another common stressor, leads to a marked upregulation of CD73 on melanoma cells in a time-dependent manner, a process mediated by autocrine release of TGF-β1 and subsequent activation of the TGFβ/SMAD3 signaling pathway (71). Under these conditions, melanoma cells also release soluble, enzymatically active CD73 via matrix metalloproteinase MMP-9-mediated cleavage (69) (**Figure 2**) (**Table 1**). This soluble form can extend the immunosuppressive adenosine gradient beyond the immediate vicinity of the tumor cell membrane.

**Table 1:** Multidimensional regulation of CD73 (*NT5E*) expression in melanoma.

Regulatory layer	Factor/pathway	Molecular mechanism/context
Transcriptional	HIF-1α	Direct transactivation of *NT5E* under hypoxia
	HOXD13	Co-regulates angiogenesis and immune evasion programs
	MAPK (BRAF/MEK)	Linked to mesenchymal-like phenotype switching
Epigenetic	DNA Methylation	Hypomethylation of *NT5E* CpG island drives metastasis
	MicroRNAs	miR-155-5p (inhibition); miR-224-3p (enhancement)
Extrinsic/TME	TGF-β	SMAD3 signaling activated by nutrient stress
	TNF-α	Cooperates with MAPK via c-Jun/AP-1 complex

The functional consequences of dynamically regulated CD73 expression are multifaceted, extending well beyond its canonical role in generating immunosuppressive adenosine. On melanoma cells, CD73 acts as a critical nexus linking intrinsic oncogenic programs to extrinsic immune evasion. Its expression marks the activation of a mesenchymal-like, invasive cell state (66, 71). This is functionally underpinned by CD73’s non-enzymatic role as an adhesion receptor. Specifically, CD73 interacts with tenascin C (TnC) in the extracellular matrix, activating focal adhesion kinase (FAK) and enhancing cell migration and invasion, thereby directly regulating melanoma progression independent of its ectonucleotidase activity (72). *In vivo*, even low levels of CD73 expression on tumor cells promote B16F10 melanoma growth, angiogenesis, macrophage infiltration, and metastasis in CD73-deficient hosts (73). This pro-angiogenic role involves the upregulation of factors like VEGF and basic fibroblast growth factor (bFGF) and the activation of the MAPK signaling pathway in endothelial cells (73).

The primary immunological consequence of CD73 activity is the generation of extracellular adenosine, which engages primarily the A2ARs and A2BRs on immune cells. Signaling through the A2AR on CD8+ T cells and tumor-infiltrating lymphocytes (TILs) induces a state of functional and metabolic suppression (74). This is mediated via the PKA/mTORC1 pathway, leading to impaired cytokine production, cytotoxicity, and metabolic fitness, characterized by reduced glucose uptake and mitochondrial respiration (74, 75). Adenosine signaling also weakens the stability of cytotoxic T lymphocyte (CTL) contact with target cells, dampening serial killing ability (76). Beyond direct T cell suppression, adenosine promotes the expansion and function of Tregs and MDSCs, further cementing the immunosuppressive milieu (77, 78). Notably, specific Treg subsets defined by CD39 and CD73 expression (e.g., CD39+ CD73+ inducible Tregs) are increased in the peripheral blood, sentinel lymph nodes, and TILs of melanoma patients, highlighting adenosine generation as a novel immune escape mechanism (77).

This complex functional repertoire positions CD73 as a significant prognostic factor, although its interpretation requires careful contextualization based on cellular source. In metastatic melanoma, CD73 expression on tumor cells is detected in over half of cases and is significantly associated with decreased overall survival from biopsy (79). Conversely, CD73 expression on tumor-infiltrating mononuclear cells is associated with improved overall survival, suggesting that immune cell-derived CD73 may reflect a more immunologically active, albeit suppressed, TME (79). This dichotomy underscores that the net biological impact of the adenosinergic pathway depends on the balance and spatial distribution of its cellular components within the ecosystem.

Furthermore, CD73 expression is dynamically modulated in response to therapy, representing an adaptive resistance mechanism. In mouse models of T-cell immunotherapy and in melanoma patients relapsing after adoptive T-cell transfer or immune checkpoint blockade, CD73 is upregulated in relapse lesions, which concurrently acquire a mesenchymal-like phenotype (66). Similarly, melanoma cells that develop resistance to the BRAF inhibitor dabrafenib show increased expression of both membrane-bound and sCD73 compared to their treatment-sensitive counterparts (69). This elevation of CD73 during treatment underscores its role as a resilient, inducible barrier to durable therapeutic response. The release of sCD73 under nutrient stress or from therapy-resistant cells can also create a diffuse, cell-independent immunosuppressive zone, complicating eradication efforts (69). This dynamic and context-dependent regulation underscores the adaptability of the melanoma ecosystem and highlights why static pre-treatment CD73 assessment may have limited predictive power (66). Instead, understanding the pathways that induce CD73 in response to therapy or microenvironmental stress is critical for identifying patients most likely to benefit from adenosinergic blockade and for designing rational combination strategies to prevent its upregulation as an adaptive resistance mechanism. Overall, CD73 expression in the melanoma ecosystem is not static but dynamically sculpted by a convergence of oncogenic pathways (MAPK) and environmental stressors (hypoxia, TGF-β). Despite these mechanistic insights, a critical limitation in the current methodology is the heavy reliance on static biopsy snapshots. The TME is highly heterogeneous and constantly evolving under therapeutic pressure; static sampling fails to capture the spatial and temporal plasticity of CD73 expression. Future research must overcome this shortcoming by integrating spatial transcriptomics and longitudinal sampling to accurately map how these regulatory networks rewire the tumor architecture in real time.

## 4 CD73 as a prognostic and predictive biomarker in melanoma: from soluble forms to extracellular vesicles

The dynamic and context-dependent regulation of CD73 underscores the adaptability of the melanoma ecosystem. Consequently, there is a pressing need to identify and validate robust biomarkers that can capture this complexity to guide patient stratification for adenosinergic therapies. The prognostic and predictive value of CD73 is not monolithic but varies significantly based on its cellular source, molecular form, and spatial distribution within the tumor and systemic circulation.

In primary tumor tissue, immunohistochemical analysis reveals a complex and often compartment-specific prognostic significance for CD73. In metastatic melanoma, CD73 expression is detected in tumor cells in approximately 54% of cases and is significantly associated with decreased overall survival from biopsy in adjusted analyses (79). This association of high tumoral CD73 with poor prognosis is consistent across multiple cancer types beyond melanoma, including triple-negative breast cancer (TNBC) and head and neck squamous cell carcinoma (HNSCC) (80, 81). Conversely, and critically, CD73 expression on tumor-infiltrating mononuclear cells in melanoma metastases was found to correlate with improved overall survival (79). This dichotomous prognostic impact, where CD73 on tumor cells is deleterious while CD73 on immune infiltrates may be favorable, is mirrored in other malignancies like rectal adenocarcinoma and prostate cancer, emphasizing the necessity of distinguishing cellular origin in biomarker analyses (82, 83).

Beyond the solid tumor compartment, soluble CD73 (sCD73) in the peripheral blood has emerged as a robust, accessible, and independent prognostic and predictive biomarker. Patients with metastatic melanoma exhibit significantly higher serum CD73 enzymatic activity compared to healthy donors (84). Elevated baseline levels of this serum sCD73 activity are strongly associated with non-response to anti-PD-1 monotherapy (nivolumab or pembrolizumab) (84, 85). In multivariate analyses, high serum CD73 activity serves as an independent prognostic factor for both reduced progression-free survival (PFS) and overall survival, alongside established markers like lactate dehydrogenase (LDH) and the presence of brain metastases (84). Mechanistically, this sCD73 is enzymatically active, capable of generating immunosuppressive adenosine in the circulation (69, 86). Its levels can be dynamically regulated; for instance, nutrient stress or matrix metalloproteinase (MMP)-9 activity can promote the release of sCD73 from melanoma cells (69). Furthermore, the development of resistance to targeted therapies like the BRAF inhibitor dabrafenib is accompanied by increased expression of both membrane-bound and sCD73 (69).

The landscape of circulating biomarkers extends beyond soluble enzymes to include specific immune cell subsets. The frequency of circulating CD8+ PD-1+ CD73+ T lymphocytes at baseline has been identified as a promising predictive marker in melanoma patients treated with nivolumab (87). Patients with a low percentage (<2.3%) of this subset experienced significantly better survival and were more likely to derive clinical benefit from therapy compared to those with higher frequencies (87). This finding aligns with the concept that CD73 expression on activated T cells may contribute to their functional exhaustion or suppression. Other peripheral immune parameters, such as the neutrophil-to-lymphocyte ratio (NLR), have also shown prognostic value in melanoma immunotherapy (88).

A rapidly evolving and critical dimension of CD73 biomarker research involves EVs, particularly small extracellular vesicles (sEVs) or exosomes. These nanosized vesicles are released by both tumor and normal cells into biological fluids and carry a cargo of proteins, lipids, and nucleic acids that reflect their cell of origin (89). Tumor-derived exosomes (TEX or MTEX in melanoma) have been shown to express functional ecto-nucleotidases on their surface (90, 91). Plasma-derived sEVs from melanoma patients carry enzymatically active CD73, which can suppress T-cell functions, such as interferon-γ production, in an adenosine-dependent manner (92). Strikingly, longitudinal monitoring revealed that early during anti-PD-1 monotherapy, levels of exosomal CD73 significantly increased in non-responders, while exosomal PD-L1 increased in responders (92). The adenosinergic activity on sEVs is not uniform across cancers; while elevated in melanoma, it may be comparable to healthy donors in other cancers like head and HNSCC (93). Advanced assays, such as those using N6-etheno-labeled purine substrates with high-performance liquid chromatography detection, now enable the sensitive and specific measurement of CD73 and CD39 activity on the surface of plasma sEVs, facilitating the non-invasive assessment of patient-specific adenosinergic pathways (90, 91). These assays can discriminate between cancer patients and healthy individuals and may guide personalized therapeutic decisions by identifying which ecto-nucleotidase is the dominant contributor to adenosine production in a given patient (90, 91) (**Table 2**).

**Table 2:** CD73-related biomarkers and their clinical significance in melanoma.

Biomarker modality	Target/parameter	Clinical significance	Key findings/thresholds
Tissue IHC	Tumor cell CD73	Poor Prognosis	Associated with decreased overall survival
	Immune cell CD73	Favorable Prognosis	Associated with improved survival in metastases
Liquid Biopsy	Serum sCD73 activity	Predictive	High activity predicts resistance to anti-PD-1 therapy
	Circulating CD73+ T cells	Predictive	Low frequency correlates with better OS
Exosomes	Exosomal CD73 (MTEX)	Resistance Marker	Early increase during therapy predicts treatment failure

**Figure 3:**
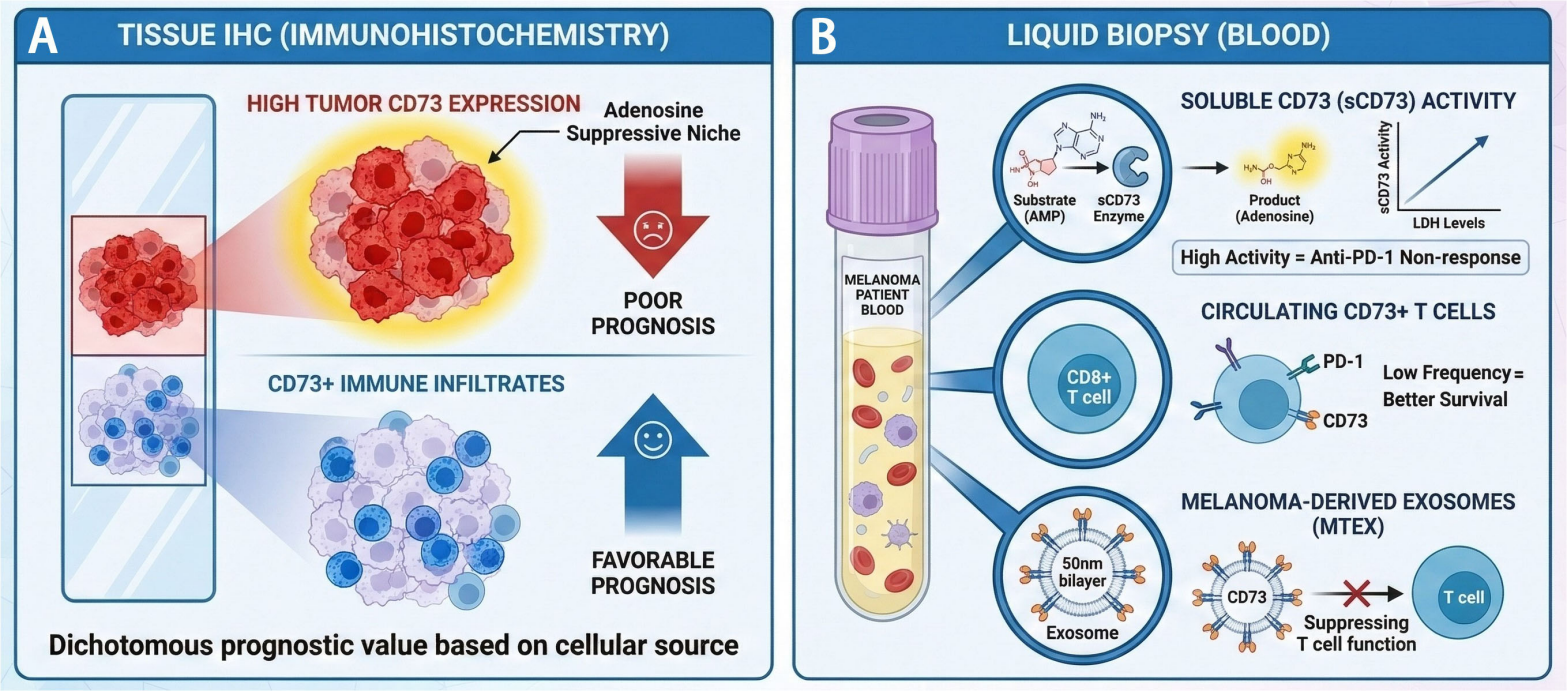
Multidimensional CD73 biomarkers for clinical prognosis and liquid biopsy-based patient stratification in melanoma. **(A)** High expression on melanoma tumor cells creates an adenosine-mediated suppressive niche, correlating with poor prognosis. Conversely, CD73 expression on immune infiltrates (e.g., tumor-infiltrating lymphocytes) is associated with favorable clinical outcomes. **(B)** A three-pronged peripheral blood assessment for non-invasive monitoring. 1) sCD73 activity: Elevated catalytic conversion of AMP to adenosine in serum correlates with high LDH levels and predicts resistance to anti-PD-1 immunotherapy. 2) Circulating CD73+ T cells: A low frequency of circulating CD8+ T cells expressing PD-1 and CD73 serves as a predictor for improved survival. 3) Melanoma-derived exosomes (MTEX): The presence of CD73-bearing exosomes in circulation facilitates systemic immunosuppression by inhibiting T-cell function.

The clinical utility of these CD73-related biomarkers lies in their potential to refine patient selection for immunotherapy and adenosinergic blockade. Combining sCD73 activity with other established biomarkers like LDH could improve prognostic stratification (84). The assessment of exosomal CD73 offers a real-time, liquid biopsy-based approach to monitor therapeutic response and the emergence of resistance mechanisms (92). Furthermore, the differential prognostic meaning of CD73 based on its cellular source within a biopsy specimen argues for the use of multiplex imaging or spatial transcriptomic techniques in future biomarker studies to fully capture this complexity (79, 80). As therapeutic targeting of CD73 advances, the integration of these biomarker modalities will be essential for identifying the patients most likely to benefit. This multidimensional biomarker approach moves beyond a binary assessment of CD73 presence or absence, instead capturing its functional activity, cellular origin, and systemic dissemination, thereby enabling a more precise application of adenosinergic therapies within the framework of precision melanoma immunotherapy (**Figure 3**). In conclusion, CD73 presents a multidimensional biomarker landscape, where its cellular compartmentalization (tumor vs. immune cells) dictates its prognostic value, and its presence in liquid biopsies (sCD73 and MTEX) offers powerful predictive potential for ICI resistance. Nevertheless, the primary shortcoming hindering the clinical translation of these liquid biomarkers is the lack of standardized isolation and quantification protocols, particularly for exosomes (MTEX). Until robust, reproducible, and scalable high-throughput assays are validated in large, prospective multicenter cohorts, the routine clinical application of CD73-related liquid biopsies will remain limited.

## 5 Therapeutic targeting of the CD73-adenosine axis: from preclinical rationale to clinical development

The multifaceted biomarker insights into CD73’s role in melanoma prognosis and immune modulation logically inform the rationale for its direct therapeutic targeting. The convergence of evidence—from its association with metastatic progression and poor survival to its functional generation of an immunosuppressive adenosine-rich niche—positions the CD73-adenosine axis as a tractable target to overcome resistance mechanisms and amplify antitumor immunity (68, 94, 95). The transition from biomarker identification to therapeutic intervention is grounded in robust preclinical models demonstrating that genetic ablation or pharmacological blockade of this pathway can reshape the TME and enhance the efficacy of established treatments (96, 97).

Foundational studies using CD73-deficient mice provided compelling evidence for the therapeutic potential of targeting this enzyme. CD73 knockout mice exhibited significantly suppressed growth of various tumors, including B16F10 melanoma, an effect dependent on CD8+ T cells and associated with increased frequency of antigen-specific T cells and IFN-γ production (96). Importantly, this protective effect was linked to both hematopoietic and non-hematopoietic expression of CD73, implicating stromal and endothelial cells in promoting tumor immune escape (96). Furthermore, CD73 deficiency conferred striking resistance to experimental metastasis, highlighting a specific role in promoting metastatic spread (96, 98). Subsequent pharmacological inhibition, using either selective small-molecule inhibitors or blocking monoclonal antibodies (mAbs), recapitulated these genetic findings, reducing tumor growth and metastasis even of CD73-negative tumors, underscoring the importance of host-derived CD73 in the TME (96). The pro-metastatic function was mechanistically linked to the suppression of NK cell maturation and cytotoxicity via signaling through the adenosine A2AR (98). These seminal works established that targeting CD73 or its downstream receptors could unleash antitumor immunity and directly impede metastatic colonization.

The strategic inhibition of the adenosinergic pathway can be approached at multiple nodes: the upstream ectonucleotidases CD39 and CD73, or the downstream adenosine receptors, primarily the high-affinity A2AR and the low-affinity A2BR. Small-molecule inhibitors of CD73, such as the potent and selective compound AB680 (quemliclustat), were designed to block the enzymatic production of adenosine (99). In preclinical settings, AB680 effectively restored T-cell proliferation, cytokine secretion, and cytotoxicity that were suppressed by CD73-derived adenosine, and it enhanced the antitumor activity of PD-1 blockade in a melanoma model (99). The discovery of novel, non-nucleotide small-molecule inhibitors with improved drug-like properties, such as compounds competing with AMP binding, further expands the chemical arsenal against CD73 (100). Simultaneously, monoclonal antibodies against CD73 have been developed, which can inhibit its function through distinct mechanisms (101, 102). Some anti-CD73 mAbs act by sterically blocking the enzyme’s catalytic site, while others, like the anti-human CD73 mAb AD2, induce clustering and internalization of CD73 from the cancer cell surface, thereby inhibiting metastasis formation independently of enzymatic blockade (101). This internalization mechanism, which can be mediated even by Fab’ fragments, points to an epitope-specific antimetastatic activity (101). Beyond singular epitope blockade, recent preclinical evidence highlights that simultaneously targeting multiple, distinct, and non-overlapping epitopes on the CD73 dimer can yield superior therapeutic outcomes. These multi-epitope targeting or oligoclonal antibody strategies not only maximize the steric hindrance of the catalytic cleft but also induce more rapid and profound CD73 clustering and intracellular degradation, ultimately leading to enhanced tumor control compared to conventional single-epitope monoclonal antibodies (103–105). For the upstream enzyme CD39, therapeutic mAbs like the fully human antibody TTX-030 have been engineered, which potently inhibit CD39 ATPase activity via an uncompetitive allosteric mechanism, preventing the initial conversion of immunostimulatory ATP to AMP (106, 107). Interestingly, drug repurposing efforts identified the ALK inhibitor ceritinib as a novel, brain-permeant small-molecule inhibitor of CD39, providing an alternative scaffold for future inhibitor development (108).

Targeting the adenosine receptors, particularly A2AR, represents a parallel and complementary strategy. Preclinical studies demonstrated that A2AR antagonists (A2ARi) or genetic deletion of host A2AR significantly reduced metastatic burden and prolonged survival in mouse models of melanoma and breast cancer (98, 109). The antimetastatic effect of A2A blockade was critically dependent on enhancing NK cell function in a perforin-dependent manner (98, 109). Beyond NK cells, adenosine signaling via A2AR on T cells directly promotes a state of anergy and can foster the generation of adaptive Tregs, thereby establishing peripheral tolerance (78). Consequently, blocking A2AR signaling can reverse T-cell exhaustion and shift the differentiation balance from precursor exhausted T cells (Tpex) towards more terminally exhausted effectors, a process implicated in the response to anti-PD-1 therapy (110). The A2BR, while less studied, also contributes to immunosuppression, particularly in contexts like pancreatic cancer where it can establish a feedforward circuit on cancer-associated fibroblasts (CAFs) to enforce the CD73 checkpoint (111). This complexity suggests that dual A2AR/A2BR antagonists or combination strategies may be required for maximal effect in certain tumor types.

Building on these mechanisms, the most compelling preclinical rationale for targeting the adenosinergic pathway lies in its synergistic potential with other therapies, especially ICIs. The combination of an A2AR antagonist with an anti-PD-1 mAb significantly reduced metastatic burden and prolonged survival in mice compared to either monotherapy, an effect most pronounced in tumors expressing high levels of CD73 (109). This synergy extends to combinations with anti-CTLA-4 and is mechanistically linked to enhanced NK and CD8+ T cell function (109). Similarly, the CD73 inhibitor AB680 combined with anti-PD-1 showed enhanced antitumor activity (99). This combinatorial benefit is not limited to immunotherapy; adenosinergic blockade also synergizes with targeted therapies. In BRAF-mutant melanoma, combined inhibition of BRAF/MEK and the A2AR provided significant protection against tumor initiation and metastasis, and interestingly, targeted therapy itself was found to downregulate tumor cell CD73 expression (68). Furthermore, adenosinergic inhibition can potentiate the effects of chemotherapy and radiotherapy. For instance, a nanoliposome co-delivering oxaliplatin (an immunogenic cell death inducer) and the CD39 inhibitor POM-1 was designed to simultaneously stimulate ATP release and prevent its degradation to adenosine, leading to potent antitumor effects in melanoma models (112). In a rectal cancer model, blocking CD73 enhanced both the local and abscopal effects of radiotherapy, correlating with increased IFN-γ production and T-cell cytotoxicity (113).

The translation of these robust preclinical findings into clinical development is actively underway, marking a new phase in immuno-oncology. Several A2AR antagonists, such as ciforadenant and AZD4635, have entered clinical trials. A phase I study of AZD4635, as monotherapy or combined with the PD-L1 inhibitor durvalumab, demonstrated manageable safety and preliminary signs of activity, particularly in metastatic castration-resistant prostate cancer (mCRPC) patients with a high blood-based adenosine gene signature (114). For CD73, the small-molecule inhibitor quemliclustat (AB680) and multiple blocking antibodies, including oleclumab (MEDI9447), are being evaluated in various solid tumors. Novel bispecific approaches are also emerging, such as dalutrafusp alfa, a bifunctional antibody that inhibits CD73 and traps TGF-β. A first-in-human phase I study of dalutrafusp alfa reported acceptable safety, complete CD73 target occupancy on immune cells, and undetectable levels of active TGF-β at higher dose levels (102). Building upon the foundational development of earlier bispecifics, the landscape of CD73-targeted bispecific antibodies is rapidly diversifying. Novel constructs are currently being evaluated to simultaneously bridge adenosinergic blockade with canonical immune checkpoints, prominently including dual CD73/PD-1 and CD73/LAG-3 targeting bispecific antibodies (currently under clinical investigation in trials NCT06166888 and NCT06691360, respectively). Additionally, the preclinical development of tumor-targeted bispecifics, such as anti-CD73/EGFR antibodies, offers a compelling strategy to tether CD73 inhibition directly to oncogene-driven tumor cells, thereby maximizing target-site drug retention while minimizing systemic off-target toxicities. Clinical investigation of CD39 inhibition is more nascent, with agents like TTX-030 in early-phase trials. The clinical experience so far reinforces the biomarker-driven hypothesis; for example, the efficacy of the A2ARi and anti-PD-1 combination in preclinical models was contingent on high tumor CD73 expression, suggesting that patient stratification using CD73 as a predictive biomarker will be crucial for clinical success (109) (**Table 3**). Furthermore, the exploration of adenosinergic inhibitors is expanding into novel delivery modalities and combination regimens, including with nanoplatforms designed to remodel the hypoxic, immunosuppressive TME (115–117). It is important to note that, to date, no therapeutic agents targeting the CD39/CD73/adenosine receptor axis have been approved by the FDA for melanoma or any other malignancies; all aforementioned drugs remain strictly under clinical investigation. To summarize, robust preclinical data have successfully translated the rationale of targeting the CD73-adenosine axis into a diverse arsenal of clinical-stage therapeutics, ranging from small-molecule inhibitors to monoclonal and bispecific antibodies. However, a significant shortcoming in current drug development is the incomplete understanding of systemic on-target/off-tumor effects. Given the physiological importance of adenosine in cardiovascular and neurological homeostasis, achieving the delicate balance between adequate intratumoral drug penetration and the minimization of systemic toxicity remains an unresolved challenge that requires more sophisticated pharmacokinetic profiling.

**Table 3:** Therapeutic agents targeting the CD73-adenosine axis in clinical trials.

Target	Agent name	Modality	Clinical potential/context	Mechanism of action
CD73	Oleclumab (MEDI9447)	Monoclonal Antibody	Evaluated in solid tumors	Steric enzymatic blockade
	AB680 (Quemliclustat)	Small Molecule	Combined with anti-PD-1	Potent, selective reversible inhibition
	Dalutrafusp alfa	Bispecific Antibody	First-in-human phase I	Dual CD73 inhibition and TGF-β trapping
CD39	TTX-030	Monoclonal Antibody	Early-phase trials	Uncompetitive allosteric inhibition
A2AR	Ciforadenant/AZD4635	Small Molecule Antagonist	Phase I studies	Blockade of adenosine-mediated cAMP signaling

## 6 Synergistic strategies: combining adenosinergic inhibition with established and emerging melanoma therapies

Given the multifaceted immunosuppression orchestrated by the CD73-adenosine axis and its interplay with established resistance mechanisms in melanoma, therapeutic strategies inhibiting this pathway are unlikely to succeed as standalone agents for most patients. The future of adenosinergic targeting lies in its rational integration with current standard-of-care and emerging therapeutic modalities. Preclinical and early clinical data robustly support the concept that disrupting adenosine signaling can remove a critical brake on antitumor immunity, thereby amplifying the efficacy of various treatment approaches, from immune checkpoint blockade (ICB) and targeted therapy to radiotherapy, chemotherapy, and adoptive cell therapies (68, 97, 109).

The most clinically advanced and mechanistically intuitive synergistic strategy involves combining adenosinergic inhibitors with ICB, particularly anti-PD-1/PD-L1 monoclonal antibodies. The mechanistic synergy is clear: while PD-1 blockade aims to reinvigorate exhausted T cells in the TME, adenosine signaling through the A2AR directly induces T cell dysfunction and exhaustion (118). Preclinical studies in melanoma models have demonstrated that the combination of an A2AR antagonist with an anti-PD-1 antibody significantly reduces metastatic burden and prolongs survival compared to either monotherapy (109). This effect was critically dependent on high tumor CD73 expression and involved NK cells and IFN-γ, highlighting a biomarker-driven and multimodal immune mechanism (98, 109). Similarly, blockade of CD39 or CD73 enhances the efficacy of anti-PD-1/PD-L1 therapy by preventing the degradation of immunostimulatory ATP into immunosuppressive adenosine, thereby preserving a pro-inflammatory milieu (119, 120). Clinical translation of this rationale is ongoing, with trials combining A2AR antagonists like ciforadenant or AZD4635 with anti-PD-(L)1 agents showing preliminary signs of activity (114, 121). Beyond PD-1/PD-L1, combinations with anti-CTLA-4 are also promising. While the primary mechanism of anti-CTLA-4 involves blocking inhibitory signals on effector T cells, evidence suggests that antibodies of certain isotypes (e.g., IgG2a) can also deplete intratumoral Tregs via Fcγ receptor-mediated antibody-dependent cellular cytotoxicity (ADCC) (122). Since Tregs are a major source of CD39 and CD73 in the TME, their depletion could concurrently reduce adenosinergic suppression. An afucosylated anti-CD39 antibody designed to enhance ADCC was shown to deplete immunosuppressive cells, including Tregs and tumor-associated macrophages, and synergize with anti-PD-L1 therapy in mouse models (119). This approach of coupling enzymatic inhibition with immune cell depletion represents a potent combinatorial strategy.

For patients with BRAF-mutant melanoma receiving MAPK pathway inhibitors (BRAFi/MEKi), adenosinergic blockade offers a complementary approach to overcome microenvironmental resistance. Targeted therapy with dabrafenib and trametinib was found to downregulate CD73 expression on melanoma cells, yet the residual adenosine pathway activity continues to promote an immunosuppressive TME (68). Combining BRAFi/MEKi with A2AR inhibition provided significant protection against tumor initiation and metastasis in preclinical models, suggesting that adenosine blockade can enhance the immunomodulatory effects of targeted therapy (68). Furthermore, the CD39 inhibitor POM-1 demonstrated efficacy in suppressing metastases when combined with BRAFi/MEKi, underscoring the potential of targeting the upstream node of the adenosinergic pathway in this context (120).

Radiotherapy (RT) and certain chemotherapeutic agents can induce immunogenic cell death (ICD), characterized by the release of damage-associated molecular patterns like ATP, which acts as a “find-me” signal for immune cells (123). However, this immunostimulatory ATP is rapidly hydrolyzed to adenosine by highly expressed CD39 and CD73 in the TME, paradoxically amplifying immunosuppression (112). Therefore, combining ICD-inducing therapies with adenosinergic inhibitors is a logical strategy to convert the TME from immunosuppressive to immunogenic. In a murine rectal cancer model, RT increased intratumoral CD73 expression and adenosine levels; combining RT with an anti-CD73 antibody enhanced local control and induced abscopal effects on non-irradiated metastases, associated with boosted T cell functionality (113). Similar synergy was demonstrated using a nanoplatform that combined a high-Z radiosensitizer with a CD73 inhibitor, which enhanced RT-induced *in situ* vaccination and systemic antitumor immunity (124). Chemotherapy-induced ICD can also be leveraged. A liposomal nanoplatform co-delivering the ICD inducer oxaliplatin and the CD39 inhibitor POM-1 was designed to simultaneously stimulate immunity (via ATP release) and relieve immunosuppression (by blocking ATP-to-adenosine conversion), resulting in potent antitumor effects and long-term immune memory in melanoma models (112). Another innovative approach involves an oncolytic hydrogel that co-delivers an ICD inducer and a CD73 inhibitor (AMPCP); this combination, when paired with anti-PD-L1, achieved complete tumor regressions in melanoma-bearing mice and induced lasting systemic immunity (117).

The success of adoptive cell therapies, such as chimeric antigen receptor (CAR) T cells, in solid tumors like melanoma is limited by the immunosuppressive TME, where adenosine is a key mediator. A2AR signaling directly impairs CAR T cell function, suppressing their proliferation, cytokine production, and cytotoxic capacity (59). Genetic strategies to knock down A2AR expression in mesothelin-targeting CAR T cells rendered them resistant to adenosine-mediated suppression (59). An alternative approach involves disrupting the intracellular localization of PKA, a key mediator of adenosine and prostaglandin E2 signaling. Engineering CAR T cells to express a peptide that inhibits PKA-ezrin interaction enhanced their effector function, improved their trafficking into tumors, and increased their antitumor efficacy in the presence of adenosine (125). These studies validate adenosinergic pathway inhibition as a crucial engineering strategy to fortify cellular therapies against the hostile TME. Furthermore, novel cellular engineering strategies are emerging to metabolically armor CAR T cells against the hostile purinergic TME. A highly innovative approach involves engineering CAR T cells to overexpress adenosine deaminase (ADA). This modification equips the engineered T cells with the intrinsic ability to rapidly catabolize immunosuppressive adenosine into inosine—a metabolite that is functionally inert or potentially even immunostimulatory—thereby preserving CAR T cell expansion and effector functions in adenosine-rich solid tumors (126).

Emerging nanotechnology platforms offer sophisticated means to implement synergistic adenosinergic targeting by enabling spatiotemporally controlled co-delivery of multiple therapeutic agents. These biomimetic nanoplatforms can be designed to tackle the adenosinergic axis at multiple levels. For instance, nanoparticles camouflaged with cancer cell membranes can achieve homologous tumor targeting and co-deliver oxygen-generating catalase and CD73 siRNA (116). This approach simultaneously alleviates tumor hypoxia (a major driver of HIF-1α-mediated CD73 upregulation) and silences CD73 expression, leading to enhanced PD-L1 blockade efficacy (116). Another nanoplatform co-delivered the CRISPR/Cas9 system for PD-L1 knockout and manganese dioxide nanoparticles to relieve hypoxia and disrupt the hypoxia-CD39/CD73-adenosine axis, achieving combined chemodynamic and immunotherapy (115). These multifunctional systems exemplify the next generation of combination therapies that rationally remodel the physical, metabolic, and immunosuppressive barriers of the TME in a coordinated manner.

**Figure 4:**
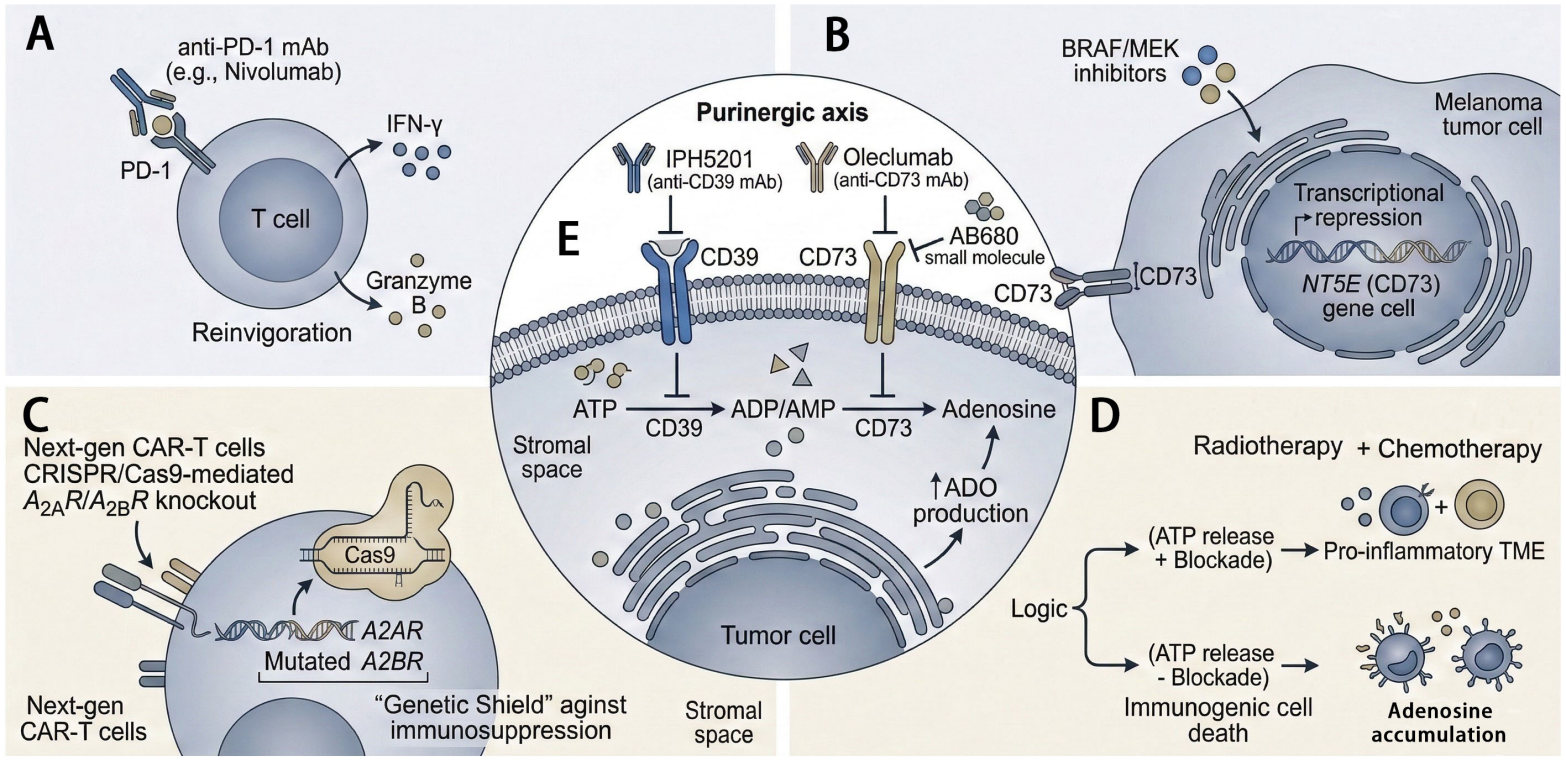
Therapeutic synergies for dismantling the adenosinergic barrier in the melanoma tumor microenvironment. **(A)** Immune checkpoint blockade using anti-PD-1 mAbs promotes T-cell reinvigoration, characterized by increased secretion of IFN-γ and Granzyme B **(B)** BRAF/MEK inhibitors exert synergistic effects by inducing transcriptional repression of the *NT5E* (CD73) gene in melanoma tumor cells. **(C)** Next-generation CAR-T cells are engineered with CRISPR/Cas9-mediated A2AR/A2BR knockout, providing a “genetic shield” that maintains effector function against adenosine-mediated suppression. **(D)** Immunogenic cell death (ICD) induced by radiotherapy and chemotherapy releases ATP; however, the pharmacological blockade of the CD39/73 axis is required to prevent adenosine accumulation and ensure a pro-inflammatory TME. **(E)** The enzymatic conversion of ATP to adenosine is targeted by specific inhibitors, including IPH5201 (anti-CD39), Oleclumab (anti-CD73), and AB680 (small molecule inhibitor), effectively reducing the TME adenosine burden.

The integration of adenosinergic inhibitors into multimodal treatment regimens is further supported by their potential to modulate specific immune cell subsets predictive of response. For example, a high proportion of CD39+ tumor-resident memory CD8+ T cells (Trm) in baseline melanoma specimens is associated with improved recurrence-free survival after adjuvant anti-PD-1 therapy (127). This suggests that therapies which enhance or preserve this population could be beneficial. Since chronic antigen exposure can lead to increased CD39 expression on T cells as a feedback mechanism (128), the interplay between adenosinergic inhibition and the maintenance of functional CD39+ Trm populations warrants further investigation. Bispecific molecules like dalutrafusp alfa, which inhibit CD73 and trap TGF-β, represent another layer of combinatorial logic, as TGF-β is a potent inducer of CD39 and CD73 expression on MDSCs and other stromal cells (102, 129). By concurrently neutralizing these two synergistic immunosuppressive pathways, such agents may more effectively reprogram the TME. As the clinical development of these diverse combination strategies progresses, the imperative for robust predictive biomarkers, particularly tumor CD73 expression and adenosine-related gene signatures, becomes increasingly critical to identify patients most likely to benefit from adding adenosinergic blockade to their therapeutic regimen (109, 114) (**Figure 4**). In brief, rational combinatorial strategies—pairing adenosinergic inhibitors with ICB, targeted therapies, ICD-inducing chemo/radiotherapy, or CAR-T cells—represent the most promising path forward to comprehensively dismantle the immunosuppressive TME. The foremost limitation in current combinatorial research, however, is determining the optimal sequencing and dosing regimens. Concurrently targeting multiple immune pathways significantly elevates the risk of severe immune-related adverse events (irAEs). Furthermore, the field currently lacks highly precise, multiplexed predictive biomarkers to identify exactly which subpopulation of patients will safely benefit from a specific combination, rendering current trial designs somewhat empirical.

## 7 Overcoming resistance: the role of the adenosinergic pathway in adaptive and acquired treatment failure

The phenomenon of treatment resistance, encompassing both primary (innate) and acquired (adaptive) forms, remains a formidable barrier to achieving durable remissions in advanced melanoma, even with modern immunotherapies and targeted agents (8). The capacity of the tumor and its surrounding ecosystem to dynamically adapt to therapeutic pressure underpins this clinical challenge (11). Emerging evidence firmly positions the adenosinergic pathway as a central orchestrator of these adaptive responses, contributing to resistance across multiple treatment modalities through both intrinsic tumor cell mechanisms and extrinsic immunosuppressive rewiring of the TME.

Central to this adaptive resistance is the dynamic regulation of CD73. Tumor cell plasticity, exemplified by phenotype switching between proliferative and invasive, mesenchymal-like states, is intrinsically linked to CD73 induction (66). This process is driven by oncogenic MAPK signaling and can be potently amplified by inflammatory cytokines such as TNF-α present in the TME, which cooperate through transcription factors like c-Jun/AP-1 to activate NT5E transcription (66). Consequently, relapse melanomas following T-cell-based therapies, including immune checkpoint blockade (ICB) and adoptive cell transfer, frequently exhibit an upregulation of CD73 concomitant with a mesenchymal phenotype (66). This represents a direct, adaptive resistance mechanism wherein therapy-induced inflammation paradoxically fuels the expression of an immunosuppressive enzyme, blunting the very immune response the treatment aimed to amplify. This dynamic regulation suggests that baseline tumor CD73 expression alone may have limited predictive value, as its levels can be profoundly modulated by therapy (66). Furthermore, microenvironmental stressors endemic to solid tumors, such as nutrient deprivation, act as potent inducers of CD73 expression on melanoma cells (71). Serum starvation triggers an autocrine loop involving the release of TGF-β1, which subsequently activates the TGFβ/SMAD3 signaling pathway to upregulate CD73, linking metabolic stress to adenosinergic immunosuppression and enhanced invasive capacity (71).

Beyond membrane-bound CD73, the release of soluble, enzymatically active CD73 adds another layer to the resistance paradigm. Melanoma cells can shed CD73 via matrix metalloproteinase (MMP)-9-mediated cleavage, particularly under conditions of nutrient stress (69). This soluble ectoenzyme can generate immunosuppressive adenosine remotely, potentially influencing sites distant from the primary tumor cell. Notably, the adenosinergic pathway is implicated in resistance to molecularly targeted therapies as well. While BRAF/MEK inhibitors can initially suppress CD73 expression in BRAF-mutant melanoma cells, the emergence of resistance to agents like dabrafenib is associated with a marked rebound in both membrane-bound and sCD73 (69). This aligns with historical observations linking ecto-5’-nucleotidase (CD73) activity to multidrug resistance phenotypes in other cancers, potentially through mechanisms involving altered nucleotide salvage and energy homeostasis (130). The TME further amplifies this adenosinergic shield. Hypoxia, a hallmark of progressing tumors, stabilizes HIF-1α, which drives the expression of CD39 and CD73, creating a feed-forward loop for adenosine generation (70). MDSCs within the TME are particularly susceptible to this regulation; tumor-derived TGF-β can upregulate CD39 and CD73 on MDSCs via mTOR-HIF-1α signaling, enhancing their immunosuppressive and chemoprotective functions (129).

The functional consequence of elevated adenosine generation is the direct suppression and metabolic impairment of TILs. CD8+ T cells encountering chronic antigen and adenosine within the TME develop features of severe dysfunction or exhaustion (131). This exhausted state is characterized by impaired production of effector cytokines like IFN-γ, TNF-α, and IL-2, high co-expression of multiple inhibitory receptors such as PD-1, and a distinct transcriptional and epigenetic program (132). CD39 expression itself is a marker of this terminally exhausted CD8+ T cell subset in tumors, induced by a combination of TCR stimulation and exposure to cytokines like IL-6 and IL-27 in the TME (132). These CD39+ exhausted T cells are enzymatically active, consuming immunostimulatory ATP and contributing to the adenosine-rich milieu, thereby actively suppressing neighboring T cells (132). Critically, the TME imposes a state of persistent metabolic insufficiency on TILs, repressing mitochondrial biogenesis and function through chronic Akt signaling and loss of PGC1α (133). This leads to the accumulation of dysfunctional, depolarized mitochondria, which is not merely a consequence of activation but a driver of the exhaustion program, as enforced mitochondrial dysfunction can induce epigenetic reprogramming toward terminal exhaustion (134). The adenosinergic pathway intersects with this metabolic crisis, as signaling through the A2AR can further perturb T cell metabolism and function.

This immunosuppressive axis also extends to other immune effectors. NK cell antitumor activity is inhibited by melanoma cell-derived factors, including prostaglandin E2 and indoleamine 2,3-dioxygenase, which downregulate activating receptors like NKG2D (135). While not directly mediated by adenosine in that study, the overall immunosuppressive landscape shaped by the TME, of which adenosine is a key component, creates a barrier for innate immune effectors. Moreover, tumor-derived exosomes emerge as critical systemic mediators of resistance. Melanoma cell-derived exosomes (MTEX) in patient plasma are enriched in immunosuppressive proteins, including CD73, and can directly inhibit CD8+ T cell activation, induce apoptosis, and downregulate NKG2D on NK cells (136). Notably, early during anti-PD-1 monotherapy, an increase in exosomal CD73 levels is associated with treatment failure, suggesting that monitoring this parameter could identify adaptive resistance as it unfolds (92). These exosomes provide a decentralized mechanism for adenosinergic suppression, potentially explaining resistance in lesions with spatially heterogeneous CD73 expression.

The interplay between the adenosinergic pathway and other established resistance mechanisms is complex. For instance, a functional IFN-γ response signature in tumors is a strong correlate of response to ICB (137). IFN-γ signaling can upregulate PD-L1 on tumor cells via the JAK-STAT-IRF1 axis, a canonical adaptive resistance mechanism (138). However, chronic inflammation and IFN-γ exposure may also contribute to the phenotypic plasticity that drives CD73 expression (66). Conversely, the loss of MHC class I expression (“hard” lesions) renders tumors invisible to T cells, representing a non-inflammatory escape route that may be less directly linked to adenosinergic adaptation (139). The metabolic competition in the TME, where highly glycolytic tumor cells consume glucose, synergizes with adenosine-mediated suppression to starve T cells of critical nutrients, exacerbating their dysfunction (8). Therefore, the adenosinergic pathway does not operate in isolation but is embedded within a network of resistance mechanisms, often activated as a coordinated response to therapeutic or immune pressure.

Targeting this pathway, therefore, represents a strategic approach to overcome resistance. Blocking the A2AR can prevent the inhibition of tumor-reactive T and NK cells, an effect that has shown promise in reversing the refractory state in some cancers (121). This strategy aims to break the final common pathway of adenosine-mediated suppression. Given that CD73 expression is dynamically induced by therapy, combining CD73 or A2AR inhibitors with frontline treatments like ICB or targeted therapy could preempt this adaptive response. This is supported by preclinical evidence showing that supplemental oxygen, by alleviating hypoxia and downregulating HIF-1α-dependent CD39/CD73, can weaken the adenosine-mediated tumor protection and enhance T cell-mediated killing (70). The therapeutic goal is to shift the TME from an immunosuppressive, adenosine-rich state to one that permits sustained immune effector function, thereby overcoming a major circuit of adaptive and acquired treatment failure. Collectively, the therapy-induced upregulation of the CD73-adenosine axis serves as a resilient and central orchestrator of adaptive and acquired treatment failure in melanoma. Despite establishing this causal link, current research falls short in fully elucidating the complex crosstalk between adenosine signaling and other concurrent metabolic crises within the TME, such as glucose starvation and lipid peroxidation. The TME is a highly integrated metabolic network; studying the adenosinergic pathway in isolation limits our ability to completely overcome metabolic exhaustion in exhausted T cells. Bridging this gap is imperative for developing next-generation interventions that can durably reverse acquired resistance.

## 8 Conclusion and future perspectives

The landscape of melanoma therapy has been irrevocably transformed by immunotherapy, yet a significant proportion of patients still face primary or acquired resistance, underscoring the urgent need for novel therapeutic dimensions (5, 8, 140). The TME, particularly its metabolic and immunosuppressive features, constitutes a formidable barrier to durable antitumor immunity (7, 8). Among the numerous mechanisms orchestrating this immunosuppressive niche, the CD73-adenosine axis has emerged as a critical metabolic immune checkpoint, offering a compelling new target for precision therapy (13, 15, 25). This pathway, dynamically shaped by hypoxia and therapy-induced stress, provides a rational target to counteract adaptive resistance and reinvigorate exhausted immune responses (17, 97). Future progress in melanoma treatment will therefore hinge on the intelligent integration of adenosinergic inhibition into the broader framework of precision immuno-oncology, a concept supported by burgeoning research and clinical interest (10, 141).

The primary therapeutic objective is to reprogram the TME from an adenosine-rich, immunosuppressive state to a permissive milieu for sustained immune effector function (10, 15). Achieving this necessitates moving beyond a one-size-fits-all paradigm. The development of inhibitors targeting key nodes of the pathway—CD39, CD73, and the adenosine receptors A2AR and A2BR—provides a toolkit for nuanced intervention (24, 25, 97). For instance, targeting the upstream enzyme CD39 offers the dual benefit of reducing immunosuppressive adenosine while preserving or increasing pro-inflammatory extracellular ATP levels, potentially yielding a more profound immunostimulatory effect (24, 35). The choice of target may be informed by the specific cellular and molecular composition of a patient’s TME, which can be characterized through advanced profiling of tumor biopsies and liquid biopsies. This aligns with the overarching principle of precision medicine: to match the right therapeutic strategy to the right patient at the right time (142, 143).

Crucial to this precision approach is the identification of robust predictive biomarkers. As reviewed, CD73 expression on tumor cells or within EVs holds prognostic significance and may predict responsiveness to adenosinergic pathway blockade (13, 89). Furthermore, dynamic changes in adenosine pathway components or associated immune cell populations (e.g., MDSCs, Tregs) during therapy could serve as pharmacodynamic markers to guide treatment adjustments (144, 145). The integration of these biomarkers with established markers like tumor mutational burden or PD-L1 expression will be essential for constructing composite predictive models (8, 97). Future clinical trials must prioritize the collection of correlative biospecimens to validate these potential biomarkers and elucidate the mechanisms of action and resistance in patients, moving the field from empirical combination towards rationally designed regimens (18, 25).

The most immediate clinical pathway lies in strategic combination therapies. Preclinical evidence strongly supports combining adenosine pathway inhibitors with immune checkpoint blockade (ICB) to overcome a key non-redundant immunosuppressive mechanism (12, 25, 97). Early-phase clinical trials are exploring this synergy (10). However, combinations must be thoughtfully designed. For example, combining anti-CD73 with anti-PD-1 may be particularly effective in tumors where CD73-mediated adenosine production is a dominant resistance mechanism to single-agent ICB (25, 35). Furthermore, the potential of adenosinergic inhibitors to synergize with other modalities, such as targeted therapy (BRAF/MEK inhibitors), chemotherapy, or radiotherapy, warrants further exploration, as these treatments can alter the TME and potentially upregulate the adenosine pathway, creating a vulnerability to be exploited (8, 97, 146). The concept of “normalizing” the TME by alleviating hypoxia—a key driver of adenosine production—through supplemental oxygen or antiangiogenic agents represents another promising combinatorial avenue that could potentiate the effects of adenosinergic blockade (15, 17).

Despite the promise, significant translational challenges remain. The biological differences between murine models and humans, particularly regarding CD73 function, necessitate careful interpretation of preclinical data (147). Moreover, the redundancy within the purinergic signaling network—including alternative pathways for adenosine generation such as the CD38-ENPP1 axis—suggests that targeting a single component may be insufficient in some contexts, prompting research into dual or pan-inhibitors (18, 148). The development of safe and effective small-molecule inhibitors, such as the CD73 inhibitor AB680, expands the therapeutic arsenal beyond monoclonal antibodies and may offer advantages in terms of tissue penetration and oral bioavailability (149). Understanding the on-target/off-tumor effects of chronically modulating extracellular adenosine levels, given its role in normal tissue homeostasis and vascular function, will be critical for long-term safety (27, 148).

Looking ahead, the frontier of adenosinergic targeting extends beyond the classical ectoenzymes and receptors. Emerging research points to non-canonical regulatory mechanisms, such as miRNA-mediated control of gene expression (150, 151). Tumor-derived exosomes, which can carry functional CD73 and other immunosuppressive cargos, represent another mechanism by which the adenosine-mediated suppressive signal is disseminated systemically, contributing to pre-metastatic niche formation and systemic immune dysfunction (89, 152). Targeting these exosomal pathways or the miRNAs that regulate the adenosinergic axis could open new therapeutic avenues. Furthermore, the role of other purinergic receptors, such as P2Y12 on platelets within the TME, in supporting tumor progression hints at an even broader purinergic network influencing cancer immunity that is yet to be fully therapeutically explored (153, 154).

Despite the rapid preclinical and clinical progress, several critical “gray areas” remain to be navigated before the CD73-adenosine axis can be fully harnessed in precision immunotherapy. First, the profound spatial and temporal heterogeneity of CD73 expression within the melanoma TME challenges the predictive accuracy of conventional single-site static biopsies. As a countermeasure, integrating spatial transcriptomics with the dynamic, longitudinal monitoring of liquid biopsies (particularly sCD73 activity and MTEX) must become the standard for tracking real-time microenvironmental rewiring. Second, the redundancy of purinergic signaling presents a major therapeutic hurdle; the compensatory activation of alternative adenosine-generating routes, such as the CD38-ENPP1 pathway, often blunts the efficacy of singular CD73 blockade. The logical countermeasure necessitates the development and clinical validation of dual-targeting agents or pan-purinergic inhibitors to completely seal off metabolic escape routes. Finally, while synergistic combinations with ICIs, targeted therapies, or ICD-inducers are highly rational, determining the optimal dosing, sequencing, and mitigation of overlapping immune-related toxicities remains a significant gray area. Addressing this requires a paradigm shift away from empirical combinations towards biomarker-driven, adaptive clinical trial designs. Ultimately, illuminating these gray areas and implementing these robust countermeasures will be the decisive step in translating adenosinergic blockade from a promising metabolic concept into a cornerstone of precision melanoma immunotherapy.

## Glossary

Abbreviation: Full NameA2AR, Adenosine A2A receptor
A2BR: Adenosine A2B receptor
ADCC: Antibody-dependent cellular cytotoxicity
ADO: Adenosine
ADP: Adenosine diphosphate
AMP: Adenosine monophosphate
AP-1: Activator protein 1
ATP: Adenosine triphosphate
bFGF: Basic fibroblast growth factor
BRAFi/MEKi: BRAF/MEK inhibitors
CAF: Cancer-associated fibroblast
cAMP: Cyclic adenosine monophosphate
CAR: Chimeric antigen receptor
CRISPR: Clustered regularly interspaced short palindromic repeats
CTL: Cytotoxic T lymphocyte
CTLA-4: Cytotoxic T-lymphocyte-associated antigen 4
ERK: Extracellular signal-regulated kinase
EVs: Extracellular vesicles
FAK: Focal adhesion kinase
GPCR: G-protein coupled receptor
GPI: Glycosylphosphatidylinositol
HIF-1α: Hypoxia-inducible factor-1 alpha
HNSCC: Head and neck squamous cell carcinoma
HPLC: High-performance liquid chromatography
ICB: Immune checkpoint blockade
ICD: Immunogenic cell death
ICI: Immune checkpoint inhibitor
IFN-γ: Interferon-gamma
IHC: Immunohistochemistry
IL: Interleukin
IRF1: Interferon regulatory factor 1
JAK: Janus kinase
LDH: Lactate dehydrogenase
mAbs: Monoclonal antibodies
MAPK: Mitogen-activated protein kinase
mCRPC: Metastatic castration-resistant prostate cancer.
